# The moderating effect of physical activity on the association between screen-based behaviors and chronic diseases

**DOI:** 10.1038/s41598-022-19305-2

**Published:** 2022-09-05

**Authors:** Raphael H. O. Araujo, André O. Werneck, Luciana L. Barboza, Ellen C. M. Silva, Danilo R. Silva

**Affiliations:** 1 Graduation Program in Health Sciences, Londrina State University (UEL), Londrina, Brazil; 2 Center for Epidemiological Research in Nutrition and Health, Department of Nutrition, School of Public Health, University of São Paulo (USP), São Paulo, Brazil; 3 Postgraduate Program in Physical Education, University of Brasília (UnB), Brasília, Brazil; 4 Department of Physical Education, Federal University of Sergipe (UFS), São Cristóvão, Brazil; 5 Department of Sports and Computer Science, Universidad Pablo de Olavide (UPO), Seville, Spain; 6 Faculty of Health Sciences, Universidad Autónoma de Chile, Santiago, Chile

**Keywords:** Diseases, Health care

## Abstract

We analyzed the associations of screen-based behaviors with obesity, hypertension, and diabetes, and the moderation of different physical activity (PA) domains in these associations. We used data from the 2019 Brazilian National Health Survey, including data from 80,940 adults (mean age of 32.6 years). TV viewing, other screens (PC, tablet, and cell phone), PA domains (leisure-time, occupational, and transport) were collected via interview. Logistic regression models were used. There was a dose–response association of higher TV viewing with diabetes. Within the groups with medium and higher time spent on other screens, those with < 150 min/week in leisure-time PA increased the odds for obesity [1–2.9 h/day: OR = 1.18 (1.01, 1.39)] and hypertension [1–2.9 h/day: OR = 1.29 (1.08, 1.53); ≥ 6 h/day: OR = 1.47 (1.03, 2.09)]. Likewise, among the participants who spent ≥ 6 h/day of TV viewing, those with < 150 min/week of occupational PA presented higher odds for hypertension [OR = 1.61 (1.03, 2.53)]. In the group with higher use of other screens, < 150 min per week of occupational PA was associated with lower odds for obesity [1–2.9 h/day: OR = 0.81 (0.68, 0.97)] and hypertension [≥ 6 h/day: OR = 0.65 (0.44, 0.98)]. In conclusion, the associations of other screens with obesity and hypertension were strongest among those without leisure-time PA, while the moderator role of occupational PA was not clear.

## Introduction

Noncommunicable diseases (NCDs) are the main cause of deaths worldwide, with diseases such as diabetes and hypertension also presenting elevated disability levels^1^. Taking into account the relevance of NCDs to global health, one of the specific Sustainable Development Goals is to reduce premature mortality from NCDs by one third by 2030^2^. However, recent data showed opposite trends in some traditional risk factors for NCDs, with rising levels of obesity and diabetes, while levels of hypertension have reduced over the years^3^.

Movement behaviors, including physical activity and sedentary behavior, are recognized modifiable risk factors for NCDs^4^. The association between movement behaviors and cardiovascular diseases has been widely studied and the positive effects of physical activity and the risks associated with excessive sedentary behaviors on health are well established^5,6^. However, the manifestations of physical activity and sedentary behaviors in different domains or contexts (e.g. leisure-time, occupational, or transport) could be specifically associated with health outcomes^6,7^. For example, leisure-time physical activity is commonly presented as a protective factor against mortality and cardiovascular diseases^5^, while occupational physical activity possibly confers lower health benefits or may even be a risk factor for different outcomes^8^. Concerning the proxies of sedentary behavior, TV viewing presented a dose–response association with mortality, while PC use and time spent driving did not^9^. In addition, with a recent decrease in time spent on TV viewing, together with an increase in the time spent on emergent sedentary behaviors, identification of possible dose–response associations between different types of screen-based behaviors and health outcomes could support public health policies.

In addition, a recent discussion has been raised about the possible synergic effects of physical activity and sedentary behavior on health outcomes^7,10^. Previous studies suggested that high levels of physical activity can reduce the association between sitting time and TV viewing with mortality^7,11,12^. Despite previous evidence, there are still some gaps in the knowledge on the joint association of physical activity and sedentary behavior with health risk indicators, especially considering the domain-specificity of physical activity, given that most previous studies used cutoffs based on total physical activity^10,13^ or leisure physical activity^14^ and it is possible that different domains of physical activity could present different moderating associations.

Even though the interaction between physical activity and sedentary behavior with cardiovascular diseases occurs mainly through physiological pathways, physical activity practiced in different domains also has different physiological impacts^15,16^. While leisure-time physical activity is often practiced through more structured physical exercises, providing greater gains from a physiological point of view, such as reducing inflammation, regulating immunity, weight and body fat, among others, domains such as transport and occupational are less autonomous, the focus of activity is rarely on health outcomes, and consequently present lower health benefits^8,16,17^. Especially, occupational physical activity can be a risk for health outcomes because of several factors (e.g., low rest period, low intensity, high exposure period), which can lead to increased inflammation^8,15,16^. Thus, we aimed to analyze the dose–response association between screen-based behaviors and obesity, hypertension, and diabetes, as well as to analyze whether the associations of screen-based behaviors with these health outcomes are modified by the levels of domain-specific physical activity.

## Methods

The present study used data from the 2019 National Health Survey (in Portuguese: *Pesquisa Nacional de Saúde—PNS)*. The PNS is a national, home-based survey, conducted by the Brazilian Institute of Geography and Statistics (IBGE), in partnership with the Brazilian Ministry of Health^18^. The sample was obtained by three-stage conglomerate sampling. The first stage was formed by census tracts; the second by household; and the third by residents. Census tracts, households, and residents were randomly selected, and individuals aged ≥ 15 years could be selected for the individual interview.

In the 2019 PNS, 94,114 adults were interviewed; however, 2431 were younger than 18 years and 10,743 presented missing data. Thus, the final sample was composed of 80,940 participants. All the participants provided the informed consent to be included in the study. The 2019 PNS was approved by the National Research Ethics Committee of the National Health Council through (CONEP) No. 3.529.376 and was conducted following the principles of the Declaration of Helsinki**.**

### Exposures

#### Screen-based behavior

TV-viewing and other types of screen time were assessed through the following questions: (1) TV time: “*How many hours a day do you usually spend watching TV?*”. (2) Time spent using a computer, tablet, or smartphone was assessed by the question: “*In your free time, how many hours a day do you usually spend using a PC, tablet, or smartphone?*”. Possible answers were: < 1 h/day, 1 to < 2 h/day, 2 to < 3 h/day, 3 to < 6 h/day, ≥ 6 h/day. Based on the answers, we created the categories of < 1, 1–2.9, 3–5.9, and ≥ 6 h/day for each one. In this paper, the time spent using a computer, tablet, or smartphone was labeled as “other screens”.

### Physical activity

The data on physical activity were collected through a validated questionnaire, based on the one used by VIGITEL (Surveillance of Risk and Protection Factors for Chronic Diseases by Telephone Survey), which was previously validated in Brazil^19^, and is widely used in the literature^14^. Questions contained in this instrument enable estimation of the accumulated physical activity time in the different domains (leisure, transport, occupation).

Briefly, the leisure-time physical activity was estimated through the following questions: (1) “*How many days a week do you practice sports or physical exercise?*”; (2) “*In general, on the day that you practice sports and/or physical exercise, how many hours/minutes does it take?*”. The weekly physical activity leisure-time was obtained through the multiplication of the weekly frequency by the length of the participation. Participants who reported ≥ 150 min of leisure-time physical activity per week were categorized as active during leisure^20^. Transport physical activity was obtained through the sum of the weekly time spent in active commuting to work and the active transport to other destinations (i.e., supermarket, language course). Participants who reported ≥ 150 min of transport physical activity were categorized as active during transport^20^. Occupational physical activity was assessed through questions about weekly frequency and length of time in vigorous and moderate activities at work. Those who reported ≥ 150 min of occupational physical activity were categorized as active at work^20^. To check the robustness of our findings, we carried out a sensitivity analysis using the highest tertile of each physical activity domain as the cutoff.

### Outcomes

Obesity was estimated through the body mass index (BMI). The participants self-reported their weight and height, and obesity was identified as BMI ≥ 30 kg/m^2^. To collect information about a hypertension diagnosis, participants were asked: “*Has a physician ever given you a diagnosis of hypertension?*”. Likewise, the presence of diabetes was assessed through the question: “*Has a physician ever given you a diagnosis of diabetes?*”. The possible answers were: “yes”, “no”, or “during gestation”, and only “yes” was considered as the presence of hypertension or diabetes.

### Confounders

The confounders included in the present study were sex (female; male), age group (18–34; 35–49; 50–64; 65+), ethnicity (white; black; mixed; other), educational achievement (no formal education; less than secondary; complete secondary; complete college or more), current smoking (no; yes), alcohol consumption in the previous 30 days (no; yes), frequency of fruit consumption per week (0 days; 1–4 days; 5+ days), and soft drinks consumption (days per week). Obesity was also included as a covariate when hypertension and diabetes were outcomes.

### Statistical analysis

The descriptive analyses are presented as a percentage and their respective 95% confidence intervals (95% CI). Logistic regression models (crude and adjusted), reporting values of odds ratio, and 95% CI were created to analyze the association between different types of screen time and the outcomes. The moderation role of different physical activity domains was assessed through the inclusion of interaction terms. All the analyses accounted for sampling weights and were conducted in the software Stata 15.

## Results

Table 1 presents the sample characteristics. Of the 80,940 participants included, 54.3% were women [mean age of 32.6 (95% CI 32.5; 32.7)] and 44.4% reported having white ethnicity and 42.8% mixed ethnicity. Approximately 6.0% reported no formal education and 16.7% reported college completed. Leisure-time physical activity and transport physical activity were less prevalent among participants with obesity, hypertension, and diabetes than among their respective peers with not these diseases. Occupational physical activity was less prevalent among those with hypertension and diabetes than those with not these diseases, respectively. The prevalence of “≥ 6 h per day” of TV viewing was higher among participants with obesity, hypertension, and diabetes than among those with not these diseases, respectively. Finally, “≥ 6 h per day” was less prevalent among those with hypertension and diabetes than among their peers with these diseases, respectively.

**Table 1:** Sample characteristics (n = 80,940).

	Whole sample	Obesity		Hypertension		Diabetes	
	n = 80,940	No (n = 63,781)	Yes (n = 17,159)	No (n = 58,954)	Yes (n = 21,986)	No (n = 73,871)	Yes (n = 7,069)
	% (95% CI)	% (95% CI)	% (95% CI)	% (95% CI)	% (95% CI)	% (95% CI)	% (95% CI)
**Male**	45.7 (45.1; 46.4)	46.9 (46.2; 47.5)	41.8 (40.4; 43.1)	47.4 (46.7; 48.1)	40.9 (39.7; 42.0)	46.1 (45.4; 46.7)	42.0 (40.0; 44.0)
**Female**	54.3 (53.6; 54.9)	53.1 (52.5; 53.8)	58.2 (56.9; 59.6)	52.6 (51.9; 53.3)	59.1 (58.0; 60.3)	53.9 (53.3; 54.6)	58.0 (56.0; 60.0)
**Age group (years)**							
18–34	29.7 (29.1; 30.3)	31.6 (30.9; 32.3)	22.9 (21.7; 24.2)	38.2 (37.5; 39.0)	4.8 (4.3; 5.3)	32.1 (31.5; 32.8)	3.3 (2.6; 4.2)
35–49	29.6 (29.1; 30.2)	28.2 (27.6; 28.8)	34.6 (33.2; 35.9)	32.9 (32.2; 33.6)	20.0 (18.9; 21.0)	31.0 (30.4; 31.5)	14.9 (13.3; 16.6)
50–64	23.6 (23.1; 24.1)	22.7 (22.1; 23.3)	26.9 (25.8; 28.1)	19.6 (19.9; 20.1)	35.6 (34.5; 36.7)	22.5 (21.9; 23.0)	36.3 (34.5; 38.2)
65+	17.0 (16.6; 17.5)	17.4 (17.0; 17.9)	15.6 (14.8; 16.5)	9.3 (9.0; 9.7)	39.7 (38.6; 40.8)	14.5 (14.1; 14.8)	45.5 (43.5; 47.4)
**Ethnicity**							
White	44.4 (43.8; 45.0)	44.7 (44.0; 45.4)	43.2 (41.8; 44.6)	44.4 (43.7; 45.1)	44.4 (43.3; 45.6)	44.3 (43.7; 45.0)	45.1 (43.1; 47.1)
Black	11.3 (10.9; 11.7)	10.9 (10.5; 11.3)	12.6 (11.7; 13.5)	11.0 (10.5; 11.4)	12.2 (11.5; 13.0)	11.3 (10.9; 11.7)	11.6 (10.4; 12.9)
Mixed	42.8 (42.2; 43.4)	42.8 (42.1; 43.5)	42.8 (41.5; 44.2)	43.2 (42.5; 43.9)	41.6 (40.5; 42.8)	43.0 (42.3; 43.6)	41.3 (39.3; 43.2)
Other	1.5 (1.3; 1.7)	1.5 (1.3; 1.7)	1.3 (1.0; 1.7)	1.4 (1.2; 1.6)	1.7 (1.4; 2.0)	1.4 (1.3; 1.6)	2.1 (1.5; 2.8)
**Educational level**							
No formal education	6.0 (5.8; 6.3)	6.2 (5.9; 6.5)	5.3 (4.8; 5.8)	4.2 (3.9; 4.4)	11.4 (10.8; 12.1)	5.4 (5.1; 5.6)	12.8 (11.6; 14.1)
Less than secondary	42.2 (41.6; 42.8)	41.4 (40.7; 42.1)	44.9 (43.6; 46.3)	38.2 (37.5; 38.9)	53.7 (52.6; 54.9)	40.8 (40.2; 41.4)	57.0 (55.0; 59.0)
Complete secondary	35.1 (34.5; 35.7)	35.3 (34.7; 36.0)	34.4 (33.0; 35.7)	39.4 (38.6; 40.1)	22.7 (21.7; 23.7)	36.4 (35.8; 37.1)	20.7 (19.1; 22.4)
Complete college or more	16.7 (16.3; 17.2)	17.1 (16.6; 17.6)	15.4 (14.5; 16.5)	18.3 (17.7; 18.8)	12.2 (11.5; 13.0)	17.4 (16.9; 17.9)	9.6 (8.5; 10.7)
**Alcohol consumption in the previous 30 days**							
No	58.1 (57.5; 58.7)	57.9 (57.2; 58.6)	58.8 (57.4; 60.2)	54.9 (54.2; 55.7)	67.5 (66.4; 68.6)	56.8 (56.1; 57.4)	73.1 (71.3; 74.9)
Yes	41.9 (41.3; 42.5)	42.1 (41.4; 42.8)	41.2 (39.8; 42.6)	45.1 (44.3; 45.8)	32.5 (31.4; 33.6)	43.2 (42.6; 43.9)	26.9 (25.1; 28.7)
**Current smoking**							
No	88.1 (87.7; 88.4)	87.5 (87.0; 87.9)	90.0 (89.2; 90.9)	87.7 (87.2; 88.1)	89.2 (88.4; 89.9)	87.9 (87.5; 88.3)	89.6 (88.3; 90.8)
Yes	11.9 (11.6; 12.3)	12.5 (12.1; 13.0)	10.0 (9.1; 10.8)	12.3 (11.9; 12.8)	10.8 (10.1; 11.6)	12.1 (11.7; 12.5)	10.4 (9.2; 11.7)
**Fruit consumption (days)**							
0	10.4 (10.1; 10.8)	10.3 (9.9; 10.7)	10.9 (10.1; 11.7)	11.0 (10.5; 11.4)	8.8 (8.2; 9.5)	10.7 (10.3; 11.)	7.8 (6.8; 9.0)
1–4	43.2 (42.6; 43.8)	43.5 (42.8; 44.2)	42.1 (40.8; 43.5)	45.0 (44.3; 45.7)	37.9 (36.8; 39.0)	44.0 (43.4; 44.7)	34.3 (32.5; 36.2)
5+	46.4 (45.8; 47.0)	46.2 (45.5; 46.9)	47.0 (45.6; 48.4)	44.0 (43.3; 44.7)	53.3 (52.1; 54.4)	45.3 (44.7; 46.0)	57.8 (55.9; 59.8)
Soft drink, mean (95% CI)	1.32 (1.30; 1.35)	1.31 (1.28; 1.34)	1.36 (1.30; 1.41)	1.45 (1.42; 1.48)	0.94 (0.89; 0.98)	1.36 (1.33; 1.39)	0.89 (0.80; 0.99)
**Leisure-time physical activity**							
Inactive	72.9 (72.4; 73.5)	71.9 (71.3; 72.6)	76.6 (75.3; 77.7)	71.2 (70.5; 71.8)	78.2 (77.2; 79.2)	72.3 (71.7; 72.9)	80.0 (78.2; 81.6)
Active	27.1 (26.5; 27.6)	28.1 (27.4; 28.7)	23.4 (22.3; 24.7)	28.8 (28.2; 29.5)	21.8 (20.8; 22.8)	27.7 (27.1; 28.3)	20.0 (18.4; 21.8)
**Transport physical activity**							
Inactive	78.0 (77.5; 78.5)	77.3 (76.7; 77.9)	80.7 (79.6; 81.8)	77.7 (76.3; 77.6)	81.0 (80.1; 81.9)	77.7 (77.1; 78.2)	81.8 (80.1; 83.3)
Active	22.0 (21.5; 22.5)	22.7 (22.1; 23.3)	19.3 (18.2; 20.4)	23.0 (22.4; 23.7)	19.0 (18.1; 19.9)	22.3 (21.8; 22.9)	18.2 (16.7; 19.9)
**Occupational physical activity**							
Inactive	75.2 (74.6; 75.7)	74.9 (74.4; 75.5)	75.9 (74.6; 77.2)	72.8 (72.1; 73.4)	82.2 (81.2; 83.1)	74.2 (73.6; 74.8)	85.4 (83.8; 86.9)
Active	24.8 (24.3; 25.4)	25.1 (24.5; 25.7)	24.1 (22.8; 25.4)	27.2 (26.6; 27.9)	17.8 (16.9; 18.8)	25.8 (25.2; 26.4)	14.6 (13.1; 16.2)
**TV viewing (h per day)**							
< 1	29.2 (28.6; 29.8)	29.8 (29.2; 30.4)	27.0 (25.8; 28.3)	31.0 (30.4; 31.7)	23.7 (22.8; 24.7)	29.8 (29.2; 30.4)	22.3 (20.7; 24.1)
1–2.9	49.2 (48.5; 49.8)	49.6 (48.9; 50.3)	47.6 (46.3; 49.0)	49.2 (48.5; 49.9)	49.0 (47.8; 50.2)	49.4 (48.7; 50.0)	46.7 (44.7; 48.7)
3–5.9	15.8 (15.4; 16.3)	15.2 (14.8; 15.7)	17.9 (16.8; 19.0)	14.8 (14.3; 15.4)	18.7 (17.; 19.6)	15.4 (15.0; 15.9)	19.9 (18.4; 21.5)
≥ 6	5.8 (5.6; 6.1)	5.4 (5.1; 5.7)	7.5 (6.9; 8.2)	4.9 (4.6; 5.2)	8.6 (7.9; 9.2)	5.4 (5.1; 5.6)	11.1 (9.9; 12.3)
**Other screens (h per day)**							
< 1	45.7 (45.1; 46.3)	45.8 (45.1; 46.5)	45.1 (43.8; 46.5)	39.1 (38.4; 39.8)	65.0 (63.8; 66.1)	43.5 (42.8; 44.1)	69.8 (67.9; 71.7)
1–2.9	32.6 (32.0; 33.2)	32.6 (31.9; 33.2)	32.6 (31.3; 33.9)	35.6 (34.9; 36.3)	23.7 (22.7; 24.8)	33.7 (33.1; 34.3)	20.3 (18.6; 22.1)
3–5.9	13.5 (13.0; 13.9)	13.3 (12.8; 13.8)	14.1 (13.0; 15.3)	15.5 (14.9; 16.1)	7.4 (6.8; 8.1)	14.1 (13.6; 14.6)	6.2 (5.3; 7.3)
≥ 6	8.3 (8.0; 8.7)	8.3 (7.9; 8.8)	8.1 (7.4; 8.9)	9.8 (9.4; 10.3)	3.9 (3.5; 4.4)	8.7 (8.4; 9.1)	3.6 (2.9; 4.5)

Table 2 shows that increments in TV-viewing were associated with increases in the likelihood of presenting obesity, hypertension, and diabetes, with a dose–response association for diabetes. The time spent on other screens elevated the odds for obesity but did not elevate the odds for hypertension and diabetes.

**Table 2:** Associations of TV viewing and other screens with overweight, hypertension, and diabetes.

Variables	Obesity	Hypertension	Diabetes
	OR (95% CI)	OR (95% CI)	OR (95% CI)
**TV—viewing**			
< 1	ref	ref	ref
1–2.9	1.03 (0.96; 1.11)	1.11 (1.03; 1.19)	1.08 (0.96; 1.21)
3–5.9	1.29 (1.17; 1.42)	1.22 (1.11; 1.35)	1.28 (1.11; 1.46)
≥ 6	1.51 (1.33; 1.71)	1.47 (1.28; 1.68)	1.75 (1.48; 2.08)
**Other screens**			
< 1	ref	ref	ref
1–2.9	1.12 (1.04; 1.21)	0.91 (0.84; 0.99)	0.86 (0.76; 0.98)
3–5.9	1.29 (1.15; 1.44)	0.94 (0.83; 1.06)	0.87 (0.71; 1.06)
≥ 6	1.20 (1.05; 1.37)	0.97 (0.83; 1.14)	0.99 (0.77; 1.26)

Adjusted by sex; age group; ethnicity; educational achievement; body mass index; smoking; alcohol consumption; fruit consumption; soda consumption; leisure-time physical activity; transport physical activity; occupational physical activity; and TV viewing or other screens.

*CI* Confidence Interval.

Supplementary Table S1 shows that participants active during leisure time were less prone to be obese than the inactive participants [OR = 0.80 (0.74; 0.86)]. Likewise, when compared to inactive participants, those active in transport presented lower odds for obesity [OR = 0.82 (0.75; 0.88)]. Concerning occupation, active individuals had less probability for hypertension [OR = 0.91 (0.83; 0.99)] and diabetes [OR = 0.79 (0.69; 0.91)].

Table 3 shows that the odds for chronic diseases increased with the duration of time spent on TV viewing, especially among those inactive in leisure time. However, we did not observe multiplicative interactions between TV viewing categories and leisure-time physical activity for obesity, hypertension, or diabetes. Increases in time spent on other screens elevated the odds for obesity among those less physically active, but not for those most active. In addition, the multiplicative interaction analysis suggests that leisure physical activity seems to moderate the association of the time spent on other screens with obesity and hypertension. For instance, among those who spent 1 to 2.9 hours and 6 or more hours per day on other screens, being less active, increased the probability of hypertension [OR_1-2.9h/d_: 1.29 (1.08; 1.53); OR_≥6 h/d_: 1.47 (1.03; 2.09)].

**Table 3:** Joint associations of TV viewing and other screens with leisure-time physical activity in the association with obesity, hypertension, and diabetes.

Interaction between leisure-time physical activity and TV viewing						
TV viewing + LTPA	Obesity		Hypertension		Diabetes	
	Joint OR (95% CI)	Multiplicative OR (95% CI)	Joint OR (95% CI)	Multiplicative OR (95% CI)	Joint OR (95% CI)	Multiplicative OR (95% CI)
< 1 + 0–149	**1.28 (1.11; 1.48)**	–	1.00 (0.86; 1.15)	–	1.04 (0.81; 1.34)	–
< 1 + ≥ 150	ref	ref	ref	ref	ref	ref
1–2.9 + 0–149	**1.30 (1.14; 1.48)**	0.95 (0.80; 1.13)	1.08 (0.94; 1.24)	0.94 (0.78; 1.12)	1.13 (0.89; 1.43)	1.02 (0.76; 1.35)
1–2.9 + ≥ 150	1.06 (0.92; 1.23)	–	1.16 (0.99; 1.35)	–	1.06 (0.82; 1.37)	–
3–5.9 + 0–149	**1.59 (1.38; 1.84)**	0.96 (0.75; 1.23)	**1.21 (1.04; 1.41)**	1.00 (0.78; 1.28)	**1.33 (1.04; 1.70)**	1.03 (0.70; 1.50)
3–5.9 + ≥ 150	**1.30 (1.04; 1.63)**	–	1.21 (0.97; 1.51)	–	1.24 (0.87; 1.76)	–
≥ 6 + 0–149	**1.96 (1.65; 2.33)**	1.20 (0.87; 1.65)	**1.49 (1.24; 1.79)**	1.20 (0.86; 1.68)	**1.76 (1.35; 2.31)**	0.83 (0.49; 1.40)
≥ 6 + ≥ 150	1.28 (0.96; 1.70)	–	1.25 (0.92; 1.69)	–	**2.03 (1.24; 3.33)**	–

Interaction between leisure-time physical activity and other screens						
Other screens + LTPA	Obesity		Hypertension		Diabetes	
	Joint OR (95% CI)	Multiplicative OR (95% CI)	Joint OR (95% CI)	Multiplicative OR (95% CI)	Joint OR (95% CI)	Multiplicative OR (95% CI)
< 1 + 0–149	**1.13 (1.01; 1.26)**	–	0.86 (0.77; 0.95)	–	1.05 (0.91; 1.22)	–
< 1 + ≥ 150	ref	ref	ref	ref	ref	ref
1–2.9 + 0–149	**1.28 (1.14; 1.45)**	**1.18 (1.01; 1.39)**	0.83 (0.74; 0.94)	**1.29 (1.08; 1.53)**	0.91 (0.75; 1.10)	0.97 (0.74; 1.28)
1–2.9 + ≥ 150	0.96 (0.84; 1.11)	–	0.75 (0.65; 0.88)	–	0.89 (0.70; 1.13)	–
3–5.9 + 0–149	**1.51 (1.29; 1.76)**	1.26 (0.99; 1.62)	0.84 (0.72; 0.97)	1.21 (0.91; 1.62)	0.90 (0.70; 1.16)	0.93 (0.60; 1.45)
3–5.9 + ≥ 150	1.06 (0.85; 1.31)	–	0.80 (0.62; 1.04)	–	0.92 (0.62; 1.38)	–
≥ 6 + 0–149	**1.33 (1.13; 1.57)**	1.05 (0.80; 1.38)	0.91 (0.74; 1.11)	**1.47 (1.03; 2.09)**	1.03 (0.77; 1.39)	0.96 (0.54; 1.69)
≥ 6 + ≥ 150	1.12 (0.88; 1.42)	–	0.72 (0.53; 0.98)	–	1.02 (0.61; 1.71)	–

Adjusted by sex; age group; ethnicity; educational achievement; body mass index; smoking; alcohol consumption; fruit consumption; soda consumption; transport physical activity; occupational physical activity and TV viewing or other screens.

*CI* Confidence Interval, *LTPA* leisure-time physical activity.

Significant values are in [bold].

In general, Table 4 presents an increase in the odds for obesity and hypertension with increments in time spent watching TV among those who do not report at least 150 min per week of transport physical activity. However, no multiplicative interactions were observed. In addition, the odds for diabetes increased among those who spent 3–5.9 h per day watching TV and were less active, and 6 or more hours per day watching TV elevated the odds for diabetes, regardless of reaching 150 min per week of transport physical activity. Other screens categories were associated with increased odds for obesity among those less active, but no multiplicative interactions were found.

**Table 4:** Joint associations of TV viewing and other screens with transport physical activity in the association with obesity, hypertension, and diabetes.

Interaction between transport physical activity and TV viewing						
TV viewing + TPA	Obesity		Hypertension		Diabetes	
	Joint OR (95% CI)	Multiplicative OR (95% CI)	Joint OR (95% CI)	Multiplicative OR (95% CI)	Joint OR (95% CI)	Multiplicative OR (95% CI)
< 1 + 0–149	1.13 (0.95; 1.34)	–	1.08 (0.94; 1.25)	–	1.04 (0.82; 1.32)	–
< 1 + ≥ 150	ref	ref	ref	ref	ref	ref
1–2.9 + 0–149	1.17 (0.99; 1.38)	1.08 (0.89; 1.32)	**1.19 (1.04; 1.36)**	0.98 (0.81; 1.17)	1.14 (0.91; 1.42)	1.07 (0.80; 1.44)
1–2.9 + ≥ 150	0.96 (0.80; 1.15)	–	1.13 (0.96; 1.32)	–	1.01 (0.78; 1.32)	–
3–5.9 + 0–149	**1.46 (1.22; 1.74)**	1.20 (0.94; 1.54)	**1.33 (1.15; 1.55)**	1.03 (0.82; 1.29)	**1.37 (1.08; 1.74)**	1.06 (0.74; 1.50)
3–5.9 + ≥ 150	1.08 (0.86; 1.34)	–	1.20 (0.98; 1.46)	–	1.25 (0.90; 1.72)	–
≥ 6 + 0–149	**1.72 (1.41; 2.09)**	1.24 (0.88; 1.74)	**1.61 (1.34; 1.93)**	1.07 (0.78; 1.46)	**1.86 (1.44; 2.41)**	0.82 (0.49; 1.37)
≥ 6 + ≥ 150	1.23 (0.90; 1.68)	–	**1.39 (1.05; 1.83)**	–	**2.17 (1.34; 3.51)**	–

Interaction between transport physical activity and other screens						
Other screens + TPA	Obesity		Hypertension		Diabetes	
	Joint OR (95% CI)	Multiplicative OR (95% CI)	Joint OR (95% CI)	Multiplicative OR (95% CI)	Joint OR (95% CI)	Multiplicative OR (95% CI)
< 1 + 0–149	**1.33 (1.20; 1.48)**	–	**1.14 (1.03; 1.25)**	–	1.07 (0.93; 1.24)	–
< 1 + ≥ 150	ref	ref	ref	ref	ref	ref
1–2.9 + 0–149	**1.45 (1.29; 1.63)**	0.87 (0.72; 1.04)	1.00 (0.89; 1.12)	0.83 (0.70; 1.00)	0.92 (0.76; 1.12)	0.94 (0.70; 1.26)
1–2.9 + ≥ 150	**1.26 (1.06; 1.48)**	–	1.06 (0.90; 1.24)	–	0.91 (0.70; 1.18)	–
3–5.9 + 0–149	**1.64 (1.41; 1.91)**	0.81 (0.61; 1.07)	1.05 (0.89; 1.23)	0.92 (0.71; 1.21)	0.90 (0.70; 1.15)	0.81 (0.48; 1.36)
3–5.9 + ≥ 150	**1.53 (1.18; 1.97)**	–	1.00 (0.79; 1.27)	–	1.04 (0.64; 1.68)	–
≥ 6 + 0–149	**1.57 (1.33; 1.86)**	0.93 (0.70; 1.23)	1.14 (0.94; 1.39)	1.20 (0.82; 1.75)	1.00 (0.74; 1.37)	0.77 (0.43; 1.36)
≥ 6 + ≥ 150	1.27 (0.98; 1.65)	–	0.84 (0.60; 1.18)	–	1.22 (0.73; 2.05)	–

Adjusted by sex; age group; ethnicity; educational achievement; body mass index; smoking; alcohol consumption; fruit consumption; soda consumption; leisure-time physical activity; occupational physical activity and TV viewing or other screens.

*CI* Confidence Interval, *TPA* transport physical activity.

Significant values are in [bold].

Table 5 presents the joint associations of TV viewing and other screens with occupational physical activity for health outcomes. With few exceptions, the odds for obesity increased with the duration of time spent watching TV among less active individuals. In addition, among the participants who spent 6 or more hours per day watching TV, those less active were more likely to report hypertension [OR: 1.61 (1.03; 2.53)]. Among those less active, the odds for diabetes increased with increments in the time spent watching TV. Concerning the time spent on other screens, multiplicative interactions analysis revealed that among other screens of 1–2.9 h per day, the less active participants were less likely to be obese compared to the more active [OR: 0.81 (0.68; 0.97)]. Likewise, multiplicative interactions analysis revealed that among those who spent 6 or more hours per day on other screens, the less active were less prone to hypertension [OR: 0.65 (0.44; 0.98)].

**Table 5:** Joint associations of TV viewing and other screens with occupational physical activity in the association with obesity, hypertension, and diabetes.

Interaction between occupational physical activity and TV viewing						
TV viewing + OPA	Obesity		Hypertension		Diabetes	
	Joint OR (95% CI)	Multiplicative OR (95% CI)	Joint OR (95% CI)	Multiplicative OR (95% CI)	Joint OR (95% CI)	Multiplicative OR (95% CI)
< 1 + 0–149	0.99 (0.85; 1.16)	–	0.97 (0.84; 1.13)	–	1.05 (0.82; 1.33)	–
< 1 + ≥ 150	ref	ref	ref	ref	ref	ref
1–2.9 + 0–149	1.01 (0.87; 1.17)	1.00 (0.83; 1.21)	1.12 (0.97; 1.29)	1.19 (0.98; 1.43)	1.19 (0.94; 1.49)	1.32 (0.98; 1.78)
1–2.9 + ≥ 150	1.02 (0.86; 1.20)	–	0.97 (0.82; 1.15)	–	0.86 (0.65; 1.13)	–
3–5.9 + 0–149	**1.29 (1.09; 1.51)**	1.23 (0.97; 1.57)	**1.22 (1.04; 1.43)**	1.12 (0.88; 1.42)	**1.40 (1.10; 1.78)**	1.40 (0.94; 2.08)
3–5.9 + ≥ 150	1.05 (0.85; 1.31)	–	1.12 (0.90; 1.39)	–	0.96 (0.66; 1.39)	–
≥ 6 + 0–149	**1.49 (1.25; 1.79)**	1.25 (0.85; 1.82)	**1.51 (1.25; 1.82)**	**1.61 (1.03; 2.53)**	**1.84 (1.42; 2.39)**	0.86 (0.41; 1.83)
≥ 6 + ≥ 150	1.21 (0.85; 1.72)	–	0.96 (0.62; 1.48)	–	2.04 (0.98; 4.25)	–

Interaction between occupational physical activity and other screens						
Other screens + OPA	Obesity		Hypertension		Diabetes	
	Joint OR (95% CI)	Multiplicative OR (95% CI)	Joint OR (95% CI)	Multiplicative OR (95% CI)	Joint OR (95% CI)	Multiplicative OR (95% CI)
< 1 + 0–149	**1.15 (1.02; 1.29)**	–	**1.13 (1.01; 1.27)**	–	**1.36 (1.14; 1.61)**	–
< 1 + ≥ 150	ref	ref	ref	ref	ref	ref
1–2.9 + 0–149	**1.22 (1.08; 1.37)**	**0.81 (0.68; 0.97)**	1.05 (0.92; 1.19)	1.05 (0.87; 1.28)	1.14 (0.93; 1.40)	0.85 (0.62; 1.16)
1–2.9 + ≥ 150	**1.31 (1.12; 1.53)**	–	0.88 (0.74; 1.04)	–	0.99 (0.74; 1.33)	–
3–5.9 + 0–149	**1.44 (1.24; 1.68)**	0.92 (0.70; 1.19)	1.00 (0.84; 1.17)	0.79 (0.60; 1.04)	1.13 (0.87; 1.47)	0.78 (0.47; 1.29)
3–5.9 + ≥ 150	**1.37 (1.09; 1.74)**	–	1.12 (0.87; 1.43)	–	1.07 (0.67; 1.70)	–
≥ 6 + 0–149	**1.31 (1.11; 1.55)**	0.82 (0.62; 1.09)	0.99 (0.81; 1.20)	**0.65 (0.44; 0.98)**	1.30 (0.96; 1.76)	0.80 (0.39; 1.62)
≥ 6 + ≥ 150	**1.39 (1.08; 1.78)**	–	1.33 (0.92; 1.93)	–	1.20 (0.62; 2.32)	–

Adjusted by sex; age group; ethnicity; educational achievement; body mass index; smoking; alcohol consumption; fruit consumption; soda consumption; leisure-time physical activity; transport physical activity and TV viewing or other screens.

*CI* Confidence Interval, *OPA* occupational physical activity.

Significant values are in [bold].

Tables S2–S4 present the sensitivity analysis for the joint associations of TV viewing and other screens with leisure, transport, and occupational physical activity, respectively. In these analyses, we used the highest tertile as the cutoff for each physical activity domain, and similar patterns of associations were observed in comparison to the use of 150 min/week as cutoff.

## Discussion

We aimed to analyze the dose–response association between screen-based behaviors and obesity, hypertension, and diabetes, as well as to analyze whether the associations of screen-based behaviors with these chronic diseases are modified by the levels of domain-specific physical activity. We found that TV-viewing presented a dose–response association with diabetes. Within the groups with a higher time spent on other screens, physically active participants in leisure presented reduced odds for obesity and hypertension. Likewise, within the group with six or more hours per day of TV viewing, those with occupational physical activity presented reduced odds for hypertension. On the other hand, within the group with higher other screens use, occupational physical activity was associated with higher odds for obesity and hypertension.

Our findings are in line with previous studies showing that TV viewing presents a stronger association with negative health outcomes compared to other types of sedentary behaviors, such as sitting time^7^ and PC use^6^. Possible explanations are related to enhances in food intake during TV viewing^21,22^ and poor dietary habits^21^, such as snack consumption, which may elevate the probability for these health outcomes^23^. However, similar to the previous findings^7,9,24,25^, our findings were adjusted for a nutritional indicator (soft drinks). In fact, a previous study found that the association between TV viewing and different cardiovascular risk factors did not substantially change with the inclusion of nutritional indicators as confounders^25^. The pattern of TV viewing, with a higher length of uninterrupted behavior (lower number of sedentary breaks), which is different from the other behaviors, could be a possible explanation as it is associated with different risk factors and is closely linked to insulin release^26,27^. Another possibility is that the use of other screen-based devices could be associated with higher energy expenditure than TV viewing^28^. For example, a cell phone is a portable device which is easily accessible and commonly used in other contexts, such as on public transport or while waiting in lines. Thus, further research could identify how emerging types of sedentary behavior may be associated with health outcomes. The non-specificity of the question related to other screen-based devices could also lead to inaccuracy of the total time, which may reflect in the different associations.

Although TV viewing elevated the probability for chronic diseases, the associations seem to differ between those who reach or not at least 150 min of physical activity. Our findings agree with previous researchers who used total physical activity^6,13^ and leisure-time physical activity^14,29^ as possible effect modifiers in the association between sedentary behaviors and negative health outcomes. In addition, we extended these results, showing the role of transport and occupational physical activity in the association between screen-based behaviors and some chronic diseases.

Our analysis indicates that more time spent in TV viewing plus being inactive in transport is worse for chronic diseases than being less sedentary plus being active in transport. However, multiplicative interactions did not show statistical significance related to these associations. Thus, within the strata of 3–5.9 and ≥ 6 h per day of TV viewing, to reach 150 min/week of transport physical activity did not reduce the probability for the chronic diseases studied.

Multiplicative interaction reveals that within the group that spent six or more hours per day watching TV, participants active in occupation presented reduced odds for hypertension. As far as we are aware, no previous studies sought to analyze the moderator role of occupational physical activity in this association. Therefore, our results partially corroborate those that showed beneficial effects of occupational physical activity on health parameters^30^. On the other hand, within the group that spend six or more hours per day on other screens, those most active in occupation had higher odds for high blood pressure. We hypothesized that participants who spent six or more hours per day on other screens could have an overlapping of behaviors (e.g., using cell phone when on a bus), which may imply in a worse recovery than experienced by those who spent six or more hours per day watching TV, and consequently be linked to an increased health risk^15^. However, additional studies seeking to identify the moderation role of occupational physical activity in the association between emerging types of sedentary behavior and health outcomes are needed.

Our results showed that increments in time spent watching TV increased the odds for obesity, hypertension, and diabetes. Likewise, other screens also increased the odds of obesity. In addition, within the groups with higher screen-based behavior, more active participants presented fewer odds for health outcomes, such as obesity and high blood pressure. However, the effects of physical activity might differ between its domains, in the way that leisure physical activity showed the clearest protective effects against chronic diseases. It is important to highlight that our findings need to be interpreted with caution, especially those derived from multiplicative interaction. For example, although higher leisure-time physical activity moderated the likelihood of obesity for those in the category of 1–2.9 h per day of other screens, the effects were not significant for the category of 6 or more hours per day of other screens, while the results for 3–5.9 h per day do not indicate a conclusive inference [OR = 1.26 (0.99; 1.62)]. In addition, although the robustness of the analysis has been checked using the highest tertile as a cutoff, the use of different cutoff points may lead to small changes in the results. This is especially relevant given that there are no cutoff points well established for each domain of physical activity. Thus, additional studies are needed to analyze the moderating effect of the physical activity domains in different types and categories of sedentary behaviors.

Our study has strengths, such as the use of a nationally representative sample and the use of physical activity-specific domains as possible effect modifiers in the association between screen time and health outcomes. Furthermore, our findings appear to be consistent when other cutoffs were used to code the participants as less and more active, which reduces the risk of bias due to the cutoff used in the main analysis (150 min/week). However, notwithstanding the relevance of our study, some limitations must be mentioned. The cross-sectional design does not allow attribution of causal inference between exposure and outcomes. Weight, height, hypertension, diabetes, screen-based behaviors, and physical activity were all self-reported, which could present recall bias, especially in older adults^31^ and individuals with low education^32^. The information about morbidities is possibly conditioned to the participants' access to the health service. Studies on the validity of the questions used to measure screen time were not available. Furthermore, the question related to time spent on other screens did not contain any details about the context of use.

## Conclusion

TV-viewing showed a dose–response association with diabetes. The moderator role of occupational PA was not clear, given that to be active in occupation reduced the association between TV viewing and hypertension, but increased the association of other screens with obesity and hypertension. On the other hand, the associations of other screens with obesity and hypertension were strongest among those without leisure-time PA. The findings highlight the importance of reducing screen-time behaviors, especially TV viewing, as well as promoting leisure-time physical activity to mitigate the potential effects of excessive sedentary behaviors.

## Supplementary Information

Supplementary Tables.

## Data Availability

Data is available under open access through the link: https://www.ibge.gov.br/estatisticas/sociais/saude/9160-pesquisa-nacional-de-saude?=&t=microdados.
