# Supplementary material for: The moderating effect of physical activity on the association between screen-based behaviors and chronic diseases
Source: Sci Rep. 2022 Sep 5;12:15066. doi: 10.1038/s41598-022-19305-2 (PMC9445100; doi:10.1038/s41598-022-19305-2)
Supplement: Supplementary file 1 — Supplementary Tables. [file 41598_2022_19305_MOESM1_ESM.docx]

Table S1 - Associations of TV viewing and other screens with obesity, hypertension, and diabetes.

| Variables | Obesity | Hypertension | Diabetes |
| --- | --- | --- | --- |
|  | OR (95%CI) | OR (95%CI) | OR (95%CI) |
| *Leisure-time physical activity* |  |  |  |
| Inactive | ref | ref | ref |
| Active | 0.80 (0.74; 0.86) | 1.03 (0.95; 1.11) | 0.96 (0.85; 1.08) |
| *Transport physical activity* |  |  |  |
| Inactive | ref | ref | ref |
| Active | 0.82 (0.75; 0.88) | 0.93 (0.86; 1.00) | 0.97 (0.86; 1.09) |
| *Occupational physical activity* |  |  |  |
| Inactive | ref | ref | ref |
| Active | 0.97 (0.89; 1.05) | 0.91 (0.83; 0.99) | 0.79 (0.69; 0.91) |

Note. Adjusted by sex; age group; ethnicity; educational achievement; body mass index; smoking; alcohol consumption; fruit consumption; soda consumption; TV viewing; other screens; and leisure-time physical activity or transport physical activity or occupational physical activity. CI, Confidence Interval. The cutoff of ≥150 minutes/week of physical activity was used to code the participants as active in each physical activity domain.

Table S2 - Joint associations of TV viewing and other screens with leisure-time physical activity in the association with obesity, hypertension, and diabetes.

| Interaction between leisure-time physical activity and TV viewing | | | | | | |
| --- | --- | --- | --- | --- | --- | --- |
| TV viewing + LTPA | Obesity | | Hypertension | | Diabetes | |
|  | Joint OR (95% CI) | Multiplicative OR (95% CI) | Joint OR (95% CI) | Multiplicative OR (95% CI) | Joint OR (95% CI) | Multiplicative OR (95% CI) |
| <1 + Tertiles 1-2 | **1.17 (1.10; 1.47)** | - | 1.00 (0.87; 1.16) | - | 1.05 (0.82; 1.34) | - |
| <1 + Tertile 3 | ref | ref | ref | ref | ref | ref |
| 1-2.9 + Tertiles 1-2 | **1.29 (1.13; 1.47)** | 0.97 (0.81; 1.15) | 1.09 (0.95; 1.25) | 0.94 (0.79; 1.13) | 1.13 (0.89; 1.44) | 1.04 (0.78; 1.38) |
| 1-2.9 + Tertile 3 | 1.05 (0.91; 1.22) | - | 1.15 (0.98; 1.35) | - | 1.05 (0.81; 1.35) | - |
| 3-5.9 + Tertiles 1-2 | **1.58 (1.37; 1.83)** | 0.96 (0.75; 1.23) | **1.21 (1.04; 1.41)** | 0.99 (0.77; 1.27) | **1.33 (1.03; 1.70)** | 1.00 (0.68; 1.47) |
| 3-5.9 + Tertile 3 | **1.30 (1.04; 1.63)** | - | 1.22 (0.97; 1.53) | - | 1.26 (0.88; 1.80) | - |
| ≥6 + Tertiles 1-2 | **1.96 (1.65; 2.33)** | 1.24 (0.90; 1.71) | **1.50 (1.25; 1.81)** | 1.21 (0.86; 1.71) | **1.77 (1.35; 2.32)** | 0.84 (0.49; 1.42) |
| ≥6 + Tertile 3 | 1.25 (0.93; 1.67) | - | 1.23 (0.91; 1.68) | - | **1.02 (1.23; 3.34)** | - |
| Interaction between leisure-time physical activity and other screens | | | | | | |
| Other screens + LTPA | Obesity | | Hypertension | | Diabetes | |
|  | Joint OR (95% CI) | Multiplicative OR (95% CI) | Joint OR (95% CI) | Multiplicative OR (95% CI) | Joint OR (95% CI) | Multiplicative OR (95% CI) |
| <1 + Tertiles 1-2 | **1.13 (1.01; 1.26)** | - | **0.87 (0.78; 0.96)** | - | 1.09 (0.94; 1.27) | - |
| <1 + Tertile 3 | ref | ref | ref | ref | ref | ref |
| 1-2.9 + Tertiles 1-2 | **1.28 (1.14; 1.44)** | 1.18 (1.00; 1.38) | **0.84 (0.74; 0.95)** | **1.26 (1.06; 1.50)** | 0.93 (0.77; 1.12) | 0.91 (0.69; 1.20) |
| 1-2.9 + Tertile 3 | 0.96 (0.84; 1.11) | - | **0.76 (0.66; 0.89)** | - | 0.93 (0.73; 1.19) | - |
| 3-5.9 + Tertiles 1-2 | **1.51 (1.29; 1.76)** | 1.27 (0.99; 1.63) | **0.84 (0.72; 0.98)** | 1.21 (0.90; 1.61) | 0.92 (0.72; 1.18) | 0.87 (0.56; 1.37) |
| 3-5.9 + Tertile 3 | 1.05 (0.84; 1.30) | - | 0.80 (0.62; 1.05) | - | 0.97 (0.64; 1.45) | - |
| ≥6 + Tertiles 1-2 | **1.33 (1.13; 1.57)** | 1.06 (0.81; 1.39) | 0.92 (0.76; 1.13) | **1.51 (1.06; 2.16)** | 1.06 (0.79; 1.43) | 0.93 (0.52; 1.64) |
| ≥6 + Tertile 3 | 1.11 (0.87; 1.41) | - | **0.81 (0.51; 0.97)** | - | 1.05 (0.63; 1.76) | - |

Note. Adjusted by sex; age group; ethnicity; educational achievement; body mass index; smoking; alcohol consumption; fruit consumption; soda consumption; transport physical activity; occupational physical activity and TV viewing or other screens. CI, Confidence Interval. LTPA, leisure-time physical activity. The cutoffs for the tertile 3 among men and women were 180 and 150 minutes of moderate to vigorous physical activity per week, respectively.

Table S3 - Joint associations of TV viewing and other screens with transport physical activity in the association with obesity, hypertension, and diabetes.

| Interaction between transport physical activity and TV viewing | | | | | | |
| --- | --- | --- | --- | --- | --- | --- |
| TV viewing + TPA | Obesity | | Hypertension | | Diabetes | |
|  | Joint OR (95% CI) | Multiplicative OR (95% CI) | Joint OR (95% CI) | Multiplicative OR (95% CI) | Joint OR (95% CI) | Multiplicative OR (95% CI) |
| <1 + Tertiles 1-2 | 1.13 (0.97; 1.31) | - | 1.09 (0.96; 1.24) | - | 1.09 (0.88; 1.36) | - |
| <1 + Tertile 3 | ref | ref | ref | ref | ref | ref |
| 1-2.9 + Tertiles 1-2 | 1.16 (1.00; 1.34) | 1.02 (0.85; 1.22) | **1.19 (1.06; 1.35)** | 0.96 (0.81; 1.13) | 1.18 (0.96; 1.44) | 1.00 (0.76; 1.30) |
| 1-2.9 + Tertile 3 | 1.01 (0.86; 1.18) | - | 1.14 (0.99; 1.31) | - | 1.08 (0.85; 1.36) | - |
| 3-5.9 + Tertiles 1-2 | **1.49 (1.27; 1.75)** | **1.26 (1.01; 1.58)** | **1.34 (1.17; 1.55)** | 1.03 (0.84; 1.27) | **1.47 (1.18; 1.83)** | 1.15 (0.83; 1.59) |
| 3-5.9 + Tertile 3 | 1.04 (0.86; 1.27) | - | 1.20 (1.00; 1.43) | - | 1.17 (0.87; 1.55) | - |
| ≥6 + Tertiles 1-2 | **1.74 (1.45; 2.08)** | 1.22 (0.90; 1.67) | **1.60 (1.34; 1.89)** | 0.99 (0.72; 1.36) | **1.96 (1.54; 2.50)** | 0.89 (0.57; 1.39) |
| ≥6 + Tertile 3 | 1.26 (0.95; 1.66) | - | **1.48 (1.12; 1.97)** | - | **2.02 (1.34; 3.06)** | - |
| Interaction between transport physical activity and other screens | | | | | | |
| Other screens + TPA | Obesity | | Hypertension | | Diabetes | |
|  | Joint OR (95% CI) | Multiplicative OR (95% CI) | Joint OR (95% CI) | Multiplicative OR (95% CI) | Joint OR (95% CI) | Multiplicative OR (95% CI) |
| <1 + Tertiles 1-2 | **1.29 (1.17; 1.43)** | - | 1.09 (1.00; 1.20) | - | 1.10 (0.96; 1.25) | - |
| <1 + Tertile 3 | ref | ref | ref | ref | ref | ref |
| 1-2.9 + Tertiles 1-2 | **1.42 (1.27; 1.59)** | 0.92 (0.78; 1.08) | 0.98 (0.88; 1.10) | 0.93 (0.78; 1.10) | 0.93 (0.78; 1.12) | 0.93 (0.71; 1.22) |
| 1-2.9 + Tertile 3 | **1.20 (1.03; 1.39)** | - | 0.97 (0.83; 1.12) | - | 0.91 (0.72; 1.16) | - |
| 3-5.9 + Tertiles 1-2 | **1.57 (1.35; 1.82)** | 0.79 (0.62; 1.02) | 1.01 (0.86; 1.12) | 0.94 (0.73; 1.20) | 0.96 (0.75; 1.22) | 1.02 (0.62; 1.67) |
| 3-5.9 + Tertile 3 | **1.53 (1.23; 1.91)** | - | 0.99 (0.80; 1.22) | - | 0.86 (0.54; 1.35) | - |
| ≥6 + Tertiles 1-2 | **1.49 (1.26; 1.75)** | 0.85 (0.65; 1.10) | 1.11 (0.91; 1.35) | 1.19 (0.84; 1.68) | 1.04 (0.76; 1.41) | 0.85 (0.50; 1.45) |
| ≥6 + Tertile 3 | **1.35 (1.07; 1.71)** | - | 0.85 (0.63; 1.15) | - | 1.11 (0.69; 1.78) | - |

Note. Adjusted by sex; age group; ethnicity; educational achievement; body mass index; smoking; alcohol consumption; fruit consumption; soda consumption; leisure-time physical activity; occupational physical activity and TV viewing or other screens. CI, Confidence Interval. TPA, transport physical activity. The cutoffs for the tertile 3 among men and women were 120 and 110 minutes of moderate to vigorous physical activity per week, respectively.

Table S4 - Joint associations of TV viewing and other screens with occupational physical activity in the association with obesity, hypertension, and diabetes.

| Interaction between occupational physical activity and TV viewing | | | | | | | | | | | | |
| --- | --- | --- | --- | --- | --- | --- | --- | --- | --- | --- | --- | --- |
| TV viewing + OPA | Obesity | | | | Hypertension | | | | Diabetes | | | |
|  | Joint OR (95% CI) | | Multiplicative OR (95% CI) | | Joint OR (95% CI) | | Multiplicative OR (95% CI) | | Joint OR (95% CI) | | Multiplicative OR (95% CI) | |
| <1 + Tertiles 1-2 | 0.99 (0.84; 1.16) | | - | | 0.95 (0.81; 1.10) | | - | | 1.08 (0.85; 1.38) | | - | |
| <1 + Tertile 3 | ref | | ref | | ref | | ref | | ref | | ref | |
| 1-2.9 + Tertiles 1-2 | 1.01 (0.87; 1.18) | 1.01 (0.83; 1.23) | | 1.09 (0.94; 1.27) | | **1.24 (1.02; 1.51)** | | 1.22 (0.97; 1.54) | | 1.34 (0.98; 1.83) | |  |
| 1-2.9 + Tertile 3 | 1.01 (0.85; 1.20) | | - | | 0.93 (0.78; 1.12) | | - | | 0.84 (0.63; 1.12) | | - | |
| 3-5.9 + Tertiles 1-2 | **1.30 (1.10; 1.54)** | | **1.33 (1.04; 1.71)** | | **1.19 (1.01; 1.41)** | | 1.20 (0.93; 1.54) | | **1.43 (1.12; 1.83)** | | 1.32 (0.88; 1.98) | |
| 3-5.9 + Tertile 3 | 0.99 (0.79; 1.23) | | - | | 1.06 (0.84; 1.13) | | - | | 1.00 (0.69; 1.47) | | - | |
| ≥6 + Tertiles 1-2 | **1.48 (1.23; 1.78)** | | 1.15 (0.79; 1.69) | | **1.47 (1.21; 1.77)** | | 1.57 (1.00; 2.47) | | **1.90 (1.46; 2.47)** | | 0.89 (0.41; 1.90) | |
| ≥6 + Tertile 3 | 1.29 (0.91; 1.85) | | - | | 0.99 (0.64; 1.51) | | - | | 1.98 (0.94; 4.19) | | - | |
| Interaction between occupational physical activity and other screens | | | | | | | | | | | | |
| Other screens + OPA | Obesity | | | | Hypertension | | | | Diabetes | | | |
|  | Joint OR (95% CI) | | Multiplicative OR (95% CI) | | Joint OR (95% CI) | | Multiplicative OR (95% CI) | | Joint OR (95% CI) | | Multiplicative OR (95% CI) | |
| <1 + Tertiles 1-2 | **1.16 (1.04; 1.31)** | | - | | **1.13 (1.01; 1.27)** | | - | | **1.39 (1.16; 1.67)** | | - | |
| <1 + Tertile 3 | ref | | ref | | ref | | ref | | ref | | ref | |
| 1-2.9 + Tertiles 1-2 | **1.24 (1.10; 1.40)** | | **0.82 (0.68; 0.98)** | | 1.04 (0.91; 1.19) | | 1.03 (0.85; 1.26) | | 1.17 (0.95; 1.45) | | 0.84 (0.61; 1.16) | |
| 1-2.9 + Tertile 3 | **1.30 (1.11; 1.54)** | | - | | 0.89 (0.74; 1.07) | | - | | 1.00 (0.74; 1.36) | | - | |
| 3-5.9 + Tertiles 1-2 | **1.46 (1.25; 1.70)** | | 0.90 (0.69; 1.18) | | 1.00 (0.85; 1.19) | | 0.80 (0.61; 1.06) | | 1.15 (0.88; 1.50) | | 0.72 (0.44; 1.20) | |
| 3-5.9 + Tertile 3 | **1.39 (1.09; 1.78)** | | - | | 1.10 (0.86; 1.42) | | - | | 1.14 (0.72; 1.82) | | - | |
| ≥6 + Tertiles 1-2 | **1.32 (1.12; 1.56)** | | 0.79 (0.59; 1.05) | | 1.03 (0.84; 1.25) | | 0.77 (0.51; 1.18) | | **1.41 (1.03; 1.93)** | | 1.20 (0.67; 2.15) | |
| ≥6 + Tertile 3 | **1.44 (1.11; 1.86)** | | - | | 1.17 (0.80; 1.73) | | - | | 0.84 (0.49; 1.44) | | - | |

Note. Adjusted by sex; age group; ethnicity; educational achievement; body mass index; smoking; alcohol consumption; fruit consumption; soda consumption; leisure-time physical activity; transport physical activity and TV viewing or other screens. CI, Confidence Interval. OPA, occupational physical activity. The cutoffs for the tertile 3 among men and women were 600 and 0 minutes of moderate to vigorous physical activity per week, respectively.
